# Epigenetic landscape correlates with genetic subtype but does not predict outcome in childhood acute lymphoblastic leukemia

**DOI:** 10.1080/15592294.2015.1061174

**Published:** 2015-08-03

**Authors:** Alem S Gabriel, Fadhel M Lafta, Edward C Schwalbe, Sirintra Nakjang, Simon J Cockell, Alice Iliasova, Amir Enshaei, Claire Schwab, Vikki Rand, Steven C Clifford, Sally E Kinsey, Chris D Mitchell, Ajay Vora, Christine J Harrison, Anthony V Moorman, Gordon Strathdee

**Affiliations:** 1 Northern Institute for Cancer Research; Faculty of Medical Sciences; Newcastle University; Newcastle upon Tyne, UK; 2 Department of Applied Sciences; Northumbria University; Newcastle upon Tyne, UK; 3 Bioinformatics Support Unit; Faculty of Medical Sciences; Newcastle University; Newcastle upon Tyne, UK; 4 Department of Pediatric Haematology and Oncology; Leeds General Infirmary; Leeds, UK; 5 Department of Pediatric Oncology; John Radcliffe Hospital; Oxford, UK; 6 Department of Haematology; Great Ormond Street Hospital; London, UK

**Keywords:** 450K, ALL, biomarker, childhood, methylation, relapse

## Abstract

Although children with acute lymphoblastic leukemia (ALL) generally have a good outcome, some patients do relapse and survival following relapse is poor. Altered DNA methylation is highly prevalent in ALL and raises the possibility that DNA methylation-based biomarkers could predict patient outcome. In this study, genome-wide methylation analysis, using the Illumina Infinium HumanMethylation450 BeadChip platform, was carried out on 52 diagnostic patient samples from 4 genetic subtypes [*ETV6-RUNX1*, high hyperdiploidy (HeH), *TCF3-PBX1* and dic(9;20)(p11–13;q11)] in a 1:1 case-control design with patients who went on to relapse (as cases) and patients achieving long-term remission (as controls). Pyrosequencing assays for selected loci were used to confirm the array-generated data. Non-negative matrix factorization consensus clustering readily clustered samples according to genetic subgroups and gene enrichment pathway analysis suggested that this is in part driven by epigenetic disruption of subtype specific signaling pathways. Multiple bioinformatics approaches (including bump hunting and individual locus analysis) were used to identify CpG sites or regions associated with outcome. However, no associations with relapse were identified. Our data revealed that *ETV6-RUNX1* and dic(9;20) subtypes were mostly associated with hypermethylation; conversely, *TCF3-PBX1* and HeH were associated with hypomethylation. We observed significant enrichment of the neuroactive ligand-receptor interaction pathway in *TCF3-PBX1* as well as an enrichment of genes involved in immunity and infection pathways in *ETV6-RUNX1* subtype. Taken together, our results suggest that altered DNA methylation may have differential impacts in distinct ALL genetic subtypes.

## Introduction

Acute lymphoblastic leukemia (ALL) is the most common form of childhood cancer, representing more than 80% of diagnosed childhood leukemia cases in the UK each year, with a gradually increasing incidence.^1^ It has long been established that chromosomal abnormalities are major drivers of ALL. Current treatment involves risk stratification guided by age and white blood cell count (WBC), karyotype, and treatment response.^2,3^ Although risk stratification and multi-agent chemotherapy have achieved around 90% survival, about 10% of patients relapse.^2^ Increasing evidence supports the inclusion of additional genomic signatures generated by transcriptome, as well as copy number changes and mutations in risk stratification.^4-6^ Current efforts are focused on identifying biomarkers for predicting relapse or reducing late complications of intensified therapy.

DNA methylation is a key epigenetic modification, which occurs primarily at CpG dinucleotide sequences.^7^ CpG sites are underrepresented throughout the genome, with the exception of short stretches of DNA known as CpG islands, which are often associated with gene promoter regions.^8^ The development of cancer, including ALL, is associated with dramatic shifts in genomic DNA methylation, involving both genome wide hypomethylation and localized hypermethylation of promoter-associated CpG islands.^9^ Hypermethylation of promoter-associated CpG islands leads to gene inactivation and many important tumor suppressor genes are known to be inactivated by this mechanism.^10^ Furthermore, the comparative ease of detection and tumor specificity of CpG island hypermethylation has led to considerable interest in their potential as novel prognostic biomarkers.^11^ Such methylation based markers may help direct current therapies, such as methylation of the DNA repair gene, *MGMT*, which predicts response to therapy in glioblastoma patients,^12^ or improve patient stratification, as we have demonstrated in chronic lymphocytic leukemia.^13^

While our understanding of methylation in cancer is improving, little is known about the role of methylation changes in the development and progression of childhood ALL. However, a number of studies have provided preliminary evidence that altered patterns of DNA methylation may be associated with outcome in ALL.^14-19^ Recent advances in whole genome screening technologies have facilitated the screening of CpG sites at a genomic level, generating a more thorough view of the methylation landscape^15,20-23^ and raised the possibility of using such technologies to identify methylation based biomarkers that could be used to further improve risk stratification in ALL patients.

In the present study we applied Illumina Infinium HumanMethylation450 genome-wide methylation arrays that cover > 485,000 methylation sites, including 99% of Refseq genes as well as 96% of CpG islands^24^ to a cohort of 52 diagnostic ALL samples in a 1:1 case-control design (26 cases who subsequently relapsed and 26 controls in continuous remission) to identify novel CpG sites that may be associated with relapse in 4 major cytogenetic subgroups in BCP-ALL.

## Results

### Genome-wide patterns of DNA methylation are strongly associated with cytogenetic subgroups

**Figure 1.**
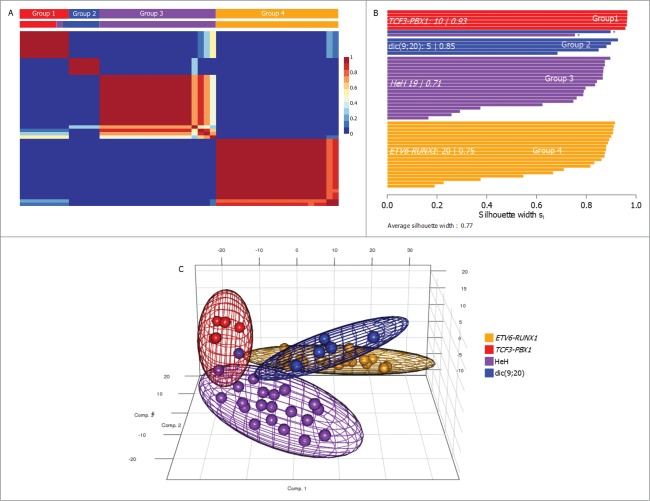
Methylation patterns identify cytogenetic groups in BCP-ALL. (**A**) Consensus clustering of DNA methylation patterns in 52 BCP-ALL samples. NMF using standard methods was carried out over 100 runs for 3–6 metagenes, with the cophenetic coefficient supporting 4 groups (metagenes). Colored squares above each column indicate the cytogenetic subgroup for each samples, showing the single *ETV6-RUNX1* samples and single dic(9;20) sample that clustered with the *TCF3-PBX1* group (group 1). (**B**) Silhouette plot by sample type and cytogenetic groups. Silhouette plots of consensus NMF subgroups demonstrate close relationships between cytogenetic subgroup and methylation subgroup assignment. For each subgroup, the number of members, the percentage of cluster members and average silhouette (s_i_) width are shown. Samples marked with an asterisk indicate outlier cytogenetic cases that do not cluster with patients of the same cytogenetic subtype. (**C**) Principal component analysis based on 10000 most variable DMCs, labeled by methylation subtype. The first 3 principal component scores are shown for each sample. For each group, covariance spheroids, colored by the predominating cytogenetic subgroup, are plotted along the 95% confidence intervals.

Genome-wide methylation data derived from the Infinium HumanMethylation450 BeadChip arrays was compared across the 52 diagnostic ALL samples and compared with control normal cells (B lymphocytes (CD19) and monocytes (CD14) derived from healthy volunteers). The majority of samples (n = 40) were derived from the 2 most common cytogenetic subtypes (*ETV6-RUNX1*, n = 20 ; HeH, n = 20), which despite having a good outcome still account for ∼40% of relapse cases due to their prevalence (Moorman et al. 2010). Additional samples from less common cytogenetic subtypes [*TCF3-PBX1*, n = 6; dic(9;20)(p11–13;q11), n = 6] were also included to determine if any relapse-associated methylation changes were independent of genetic subgroup and also to allow clearer definition of subgroup specificity of DNA methylation in childhood ALL. Non-negative matrix factorization (NMF) was used to reduce the dimensionality of the data from 10,000 probes to a few metagenes. For each factorization rank from 3 to 7, we assessed stability of factorization by cophenetic coefficient (**Fig. S1**) and silhouette scores of consensus subgroup assignments after 100 iterations (**Fig. 1A**). NMF separated the samples into 4 groups which corresponded very closely to the subgroups (**Fig. 1**), with only a single HeH sample and a single dic(9;20) sample failing to cluster with their genetic counterparts (we have excluded the possibility of cryptic *TCF-PBX1* translocation via interphase FISH). The identified subgroups are characterized by a positive silhouette score (**Fig. 1B**), indicative of samples being placed into the correct cluster, and show clear separation by principal component analysis (PCA) of the methylation data (**Fig. 1C**). This association with cytogenetic subtype is consistent with previous reports.^15,20-23^ The differentially methylated CpG sites that demonstrated subtype specificity are listed for each subtype in supplementary **Table 1**. Furthermore, there were clear differences in the genomic locations and directionality of methylation changes between the subtypes; for example, the *ETV6-RUNX1* and dic(9;20) subtypes exhibited relatively more hypermethylation than hypomethylation, while changes in the HeH and *TCF3-PBX1* subtypes were predominantly hypomethylated (**Fig. S2**). We have performed a gene enrichment pathway analysis of these subtype specific differentially methylated cytosines (DMCs) after correcting for probe distribution and multiple testing in KEGG database. Interestingly, this analysis identified 10 pathways that were significantly over-represented in the *ETV6-RUNX1* DMCs (after correction for multiple testing) and 8/10 of these pathways were related to immune function. While no pathways were identified as over-represented in the HeH and dic(9;20) subtypes, a highly significant association (*P* = 5.7 × 10^−17^, after correction) was found with neuroactive ligand-receptor interaction in the *TCF3-PBX1* subtype (online supplementary **Table 2**). The association between subtype specific DMCs and specific pathways further implicates the differential methylation in different biological behavior of the cytogenetic subtypes.

**Figure 2.**
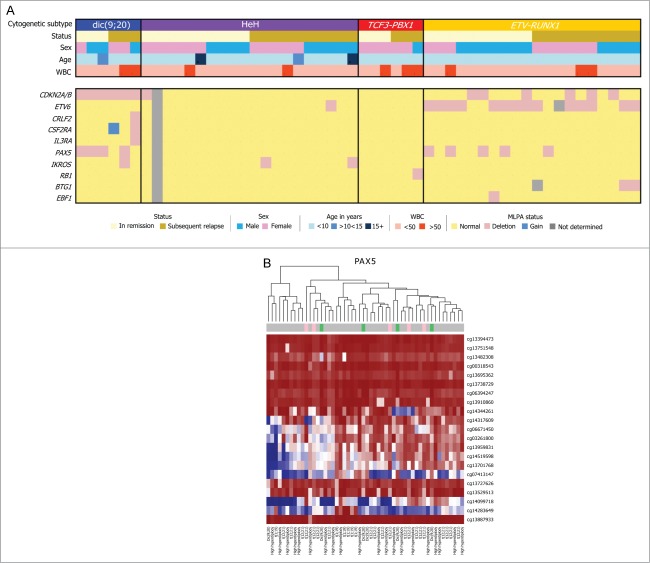
(**A**) Demographic and clinical features of 52 diagnostic bone marrow samples. Abbreviations: WBC, white blood cell; NCI risk, national cancer institute risk; SR, standard risk; HR, hazard ratio; HeH, High hyperdiploidy. Some of the gene aberrations listed are linked to the primary genetic aberrations (i.e., *CDKN2A/B* and *PAX5* in dic(9;20), rather than true focal aberrations). Similarly, gene abberations resulting from whole chromsome gain (HeH) or loss have not been shown. (**B**) Deletion of *PAX5* was observed in 5 patients via MLPA shown in pink blocks above. We clustered the data by looking at methylation probes 5 kb upstream and downstream of PAX5. The result shows that deletion status of *PAX5* does not seem to correlate with methylation values and seems independent of copy number.

All samples in the cohort had also previously been analyzed using Multiplex Ligation-dependent Probe Amplification (MLPA)^25^ to identify copy number alterations in genes with a known role in ALL development (**Fig. 2A**). It is possible that the complexity of data derived from the HumanMethylation450 BeadChip arrays was masking changes at such key leukemia associated genes or that DNA methylation changes may be acting as a second hit at sites of heterozygous deletions. Therefore, methylation at all CpG sites associated with the 8 loci (*CRLF2, IL3RA*, and *CSF2RA*, considered as *PAR1* locus) covered by the MLPA analysis were extracted from the array data. As illustrated for *PAX5* in **Figure 2B**, methylation levels were similar across the sample set. Raw methylation data could also be used to assess potential changes in copy number.^26^ This analysis confirmed the MLPA data showing *PAX5* deletions in 5 of the patients (**Fig. S3**). However, methylation of *PAX5* did not vary in the samples with confirmed deletions (**Fig. 2B**), suggesting that altered methylation was not functioning as a second hit, at least for *PAX5*.

### Genome-wide patterns of DNA methylation are not significantly different in patients that subsequently relapsed

The development of relapse is a crucial determinant of outcome, as survival rates following relapse are much lower. Identification of patients at the time of diagnosis who will subsequently relapse and, indeed, those unlikely to relapse, is extremely valuable to optimize their treatment. Consequently, genome-wide methylation data was analyzed, using a number of different methods, to attempt to discover methylation-based signatures in diagnostic samples that were predictive of subsequent relapse. As described above, unsupervised NMF consensus clustering separated the samples by underlying genetic subgroups (**Fig. 1**). However, within each subgroup, there was no evidence of clustering of samples based on eventual outcome. In case the strong association with cytogenetics was masking a relapse signature, this analysis was repeated following the removal of data from the probes that were differentially methylated between subgroups; however, this analysis also showed no evidence of clustering according to outcome and no individual CpG site exhibited a statistically significant correlation with outcome (data not shown). Furthermore, analysis of individual CpG sites [differential CpG sites were classed as having a difference in mean β value > 0.2 and an adjusted *P*-value < 0.01 (Nordlund-Backlin et al. 2013)] did not identify any individual CpG sites that was significantly associated with relapse. Genetic subtype specific analysis, for the *ETV6-RUNX1* and HeH subgroups, also yielded no individual probes significantly associated with subsequent relapse.

Altered DNA methylation often occurs coordinately across genomic regions, such as CpG islands. To determine whether any such regions were differentially methylated between samples from patients who subsequently relapsed and those who did not, a bump-hunting algorithm^27^ within the Bumphunter package was utilized, with 1000 permutations. Probes were clustered into a region based on distance: all differentially methylated probes that were located within 300 bp of another differentially methylated probe were placed into the same cluster group, so that window widths were flexible and defined by proximity and number of differentially methylated probes, rather than by fixed size. *P*-values were also adjusted to control the false discovery rate (FDR) using the Benjamini–Hochberg method. However, neither approach identified regions in which the levels of methylation were significantly different between samples from the 2 different outcome categories at 5% FDR and 10% family-wise error rate (FWER), when all samples were analyzed simultaneously (**Table 1**). This was also largely true when the HeH and *ETV6-RUNX1* groups were analyzed separately, although a weak association was found at the *EXT1* loci specifically in the HeH subgroup (**Table 1**). Genetic subtype specific analysis was again restricted to *ETV6-RUNX1* and HeH subgroups. Single gene analysis utilizing pyrosequencing validated the array-generated data at the *EXT1* locus; however, expansion of the analysis to additional diagnostic samples did not support an association between *EXT1* methylation and subsequent relapse (**Fig. S4**).

**Table 1.** Differentially methylated regions identified by Bump Hunter analysis

All samples								
Chr	Start	End	No. of CpG sites	*P*-value	FWER^1^	Width (bps)	Nearest Gene	Distance to TSS^2^
chr10	134765033	134765099	3	0.00029	0.354	67	*TTC40*	−8944
chr8	119124051	119124311	4	0.00049	0.443	261	*EXT1*	0
chr21	38468606	38468606	1	0.00042	0.750	1	*TTC3*	10516
chr2	77235218	77235218	1	0.00066	0.883	1	*LRRTM4*	514284
chr10	675888	675937	3	0.00242	0.887	50	*DIP2C*	59671
chr17	68164468	68164468	1	0.00093	0.934	1	*KCNJ2-AS1*	1075
chr9	124022933	124022933	1	0.00101	0.942	1	*GSN*	59172
chr3	168308798	168308798	1	0.00118	0.962	1	*EGFEM1P*	59276
chr6	32774788	32774788	1	0.00121	0.963	1	*HLA-DOB*	10037
chr1	248020692	248021091	4	0.00483	0.971	400	*TRIM58*	191
chr10	134765033	134765099	3	0.00029	0.354	67	*TTC40*	−8944
*ETV6-RUNX1*								
Chr	Start	End	No. of CpG sites	*P*-value	FWER	Width (bps)	Nearest Gene	Distance to TSS
chr5	158086454	158086454	1	1.64E-05	0.063	1	*EBF1*	437160
chr15	69744390	69744684	4	7.88E-05	0.236	295	*RPLP1*	−475
chr8	1651128	1651128	1	7.30E-05	0.242	1	*DLGAP2*	201596
chr4	134069593	134070441	10	0.00015	0.404	849	*PCDH10*	−29
chr5	178986620	178986906	5	0.00037	0.670	287	*RUFY1*	0
chr11	100760935	100760935	1	0.00031	0.693	1	*ARHGAP42*	202528
chr6	160023581	160024144	6	0.0009	0.898	564	*SOD2*	90209
chr21	46077454	46077731	6	0.0011	0.924	278	*TSPEAR*	53764
chr14	76015669	76015669	1	0.00079	0.946	1	*BATF*	26885
chr10	124639132	124639260	7	0.00135	0.952	129	*FAM24B*	0
HeH								
Chr	Start	End	No. of CpG sites	*P*-value	FWER	Width (bps)	Nearest Gene	Distance to TSS
chr8	119124051	119124462	5	1.58E-06	0.006	412	*EXT1*	0
chr10	675888	675937	3	0.00019	0.380	50	*DIP2C*	59671
chr16	66458043	66458043	1	0.00021	0.560	1	*BEAN1*	−3157
chr4	99850801	99851281	9	0.00047	0.625	481	*EIF4E*	505
chr22	25201958	25202163	6	0.00053	0.664	206	*SGSM1*	0
chr17	76875678	76876239	4	0.00055	0.669	562	*TIMP2*	42244
chr10	113944114	113944114	1	0.00031	0.695	1	*GPAM*	−577
chr5	150284600	150284796	2	0.00065	0.786	197	*ZNF300*	−55
chr1	248020377	248021091	8	0.00083	0.802	715	*TRIM58*	0
chr8	19459672	19460243	4	0.00089	0.807	572	*CSGALNACT1*	0
chr8	119124051	119124462	5	1.58E-06	0.006	412	*EXT1*	0

1 *P*-Value corrected for family-wise error rate, with B = 1000 permutations.

2 Transcriptional start site.

**Figure 3.**
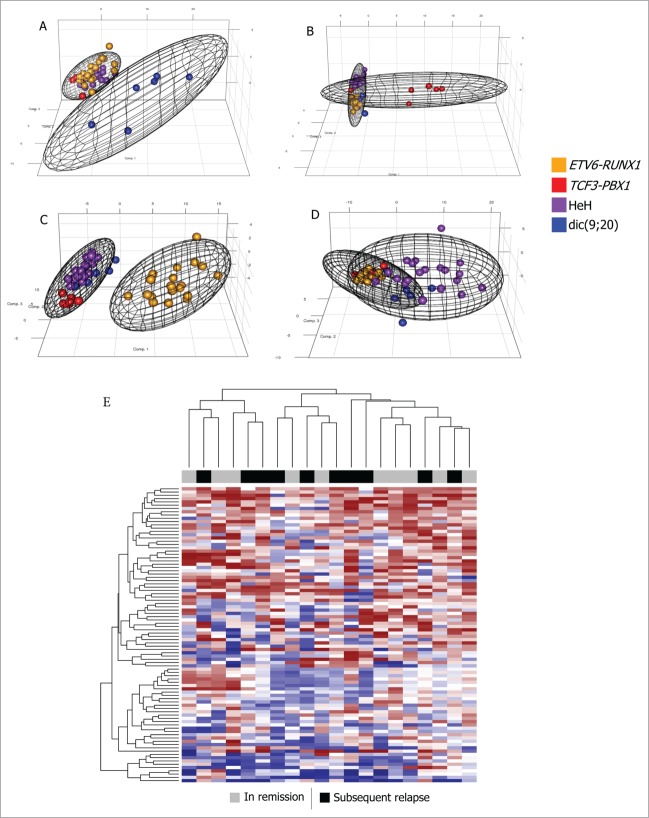
Principal component analysis of subtype-specific DMCs identified by Nordlund et al. recapitulates genetic subgroup separation and validates them as biomarkers for these subgroups. For each plot, 2 covariance spheroids have been plotted along the 95% confidence intervals for (**A**) dic(9;20) and others; (**B**) TCF3-PBX1 and others; (**C**) ETV6-RUNX1 and others; (**D**) HeH and others. Individual samples are colored by their cytogenetic status; TCF3-PBX1 cases are shown red, ETV6-RUNX1 in orange, dic(9;20) in blue and HeH in purple. (**E**) Previously reported relapse-associated DMCs in ETV6-RUNX1 positive cases are not recapitulated in our data set. The color bar at the top of the heatmap indicates sample type; continuous remission and subsequently relapsing patients are shown gray and black respectively. The heatmap shows the methylation status for 90 relapse-associated probes identified by Nordlund et al.^20^ Samples (columns) and probes (rows) were clustered using complete linkage and Euclidean distance. Fully methylated probes are shown dark red, unmethylated probes shown dark blue and hemi-methylated probes are shown in white.

A recent study by Nordlund et al.^20^ reported genome-wide methylation patterns for multiple childhood ALL cytogenetic subgroups, including the 4 included in this study. Similar to the results reported above, they also identified large-scale differences in DNA methylation between different cytogenetic subgroups. To assess the reproducibility of the genetic subgroup-specific methylation profiles identified by Nordlund et al., we determined whether the CpG marker sets identified as specific for individual cytogenetic subgroups in that study would also identify individual cytogenetic subgroups in our data set. As shown in **Figure 3**, all 4 cytogenetic subgroups were identified using their markers sets [with 54.0% (1141 / 2114), 37.7% (1136 / 3014), 28.3% (314 / 1110), 14.9% (353 / 2370) subtype specific CpG markers concordant with our analysis for *ETV6-RUNX1*, HeH, *TCF3-PBX1*, and dic(9;20) respectively, using the same criteria for defining subtype specific methylated loci].^20^

Nordlund et al. also identified a set of 90 DMCs associated with relapse, specifically in *ETV6-RUNX1* positive cases. To validate this marker set for the identification of subsequent relapse, we applied these 90 relapse predicting DMCs to our datasets. However, as shown in **Figure 3E**, unsupervised clustering using the same 90 CpG sites, did not appear to differentiate between relapse and non-relapse samples (specifically in *ETV6-RUNX1* positive cases). To determine whether single CpG sites from within this group of 90 sites were associated with relapse, each locus was assessed individually in our *ETV6-RUNX1* positive sample set. However, only one of the 90 loci exhibited a statistically significant association with subsequent relapse in our sample set (cg17033047 within the *KCNA3* locus, *P* = 0.01, uncorrected *P*-value, higher methylation levels in relapse samples). Expanding the analysis for this site to an additional 57 *ETV6-RUNX1* positive cases (of which 3 relapsed) failed to confirm the differential methylation seen in the 20 samples used for the array analysis (**Fig. S4D**). As the size of the *ETV6-RUNX1* positive sample set was small (n = 20) with only 10 relapsed *ETV6-RUNX1* cases, the possibility cannot be ruled out that weak correlations may be detectable in larger sample sets. However, it should also be noted that our data set had more relapsed *ETV6-RUNX1* cases than Nordlund et al.^20^ Taken together, these results suggest that DNA methylation at these loci is unlikely to be of significant clinical utility for the prediction of relapse in *ETV6-RUNX1* positive childhood ALL.

## Discussion

Alterations in DNA methylation are highly prevalent in childhood ALL, suggesting that they may have a major impact on the biology and clinical behavior of the disease, as well as raising the possibility that differences in DNA methylation patterns at diagnosis may be useful biomarkers for prediction of clinical outcome. In this study, genome-wide methylation analysis was carried out on 52 diagnostic ALL samples from 4 cytogenetic subgroups, in which 50% of patients subsequently relapsed and 50% remained in long-term remission. NMF consensus clustering identified multiple sub-groups within the methylation data; however, these were related to the underlying genetic differences and did not differentiate between samples with different relapse status. Different bioinformatics approaches were undertaken in an attempt to identify a single or a small number of methylation variable sites that could identify at diagnosis those patients most likely to relapse. However, none of these approaches identified any loci whose methylation status was significantly associated with subsequent relapse. Some limited evidence for an association with relapse was found at sites within the *EXT1* and *KCNA3* loci in the array data; however, neither were confirmed by pyrosequencing analysis of additional samples. The results presented here, in combination with previously published data,^20^ suggest that genome-wide methylation profiles, identified using the Infinium HumanMethylation450 BeadChip array platform, may be unlikely to yield clinically useful biomarkers for prediction of relapse in childhood ALL over and above the prognostic information already provided by cytogenetic subgroups.

However, there was a clear correlation between genome-wide patterns of DNA methylation and the different cytogenetic subgroups, consistent with previous studies.^15,20-23^ In addition, we were able to use the marker sets recently identified by Nordlund et al.,^20^ whose analysis utilized the same array platform, on our data set and validate their cytogenetic specific markers. Thus, while DNA methylation profiles did not appear to augment the prognostic information provided by standard cytogenetics, the consistency of the methylation changes in relation to cytogenetic subgroups suggests that these altered patterns of DNA methylation may be an important determinant of their different clinical behavior. Furthermore, the results suggest that genomic DNA methylation could be used as a surrogate for cytogenetic analysis in cases where genomic DNA, but no intact cells, was available.

While methylation profiles associated with cytogenetic subgroups could be validated, the marker set suggested to predict outcome in *ETV6-RUNX1* positive cases did not validate in our data set. A potential cause would be that the patients were treated on different protocols, although the treatment protocols used were highly similar.^28,29^ In addition, the patient populations were derived from different geographical locations, so differences in genetic background may have also played a role. However, the results do indicate that the identified methylation profile is not readily portable to other patient populations.

Although the cytogenetic subtypes showed a clear correlation with methylation profiles, the data presented here and previously published^20,23^ showed that this clustering was not absolute. For example, in the data set presented here, one dic(9;20) and one HeH case clustered with the *TCF3-PBX1* samples. In such cases, it is not clear whether the risk of relapse in the patients would reflect their cytogenetic subgroup or be equivalent to the subgroup defined by the methylation profile. However, the comparative rarity of these cases (only 2/52 samples in this study) meant that a much larger study would be required to have sufficient power to address the potential prognostic significance of such “outlier” samples.

While analysis of DNA methylation profiles has identified subtype specific methylation changes, it is important to note that many of the alterations identified in this and other studies are shared across cytogenetic subgroups. This implies that a large set of epigenetic changes are either a prerequisite for, or an inevitable consequence of, the development of ALL. In general, the consistency of alterations seen in ALL and indeed other tumors has led to the hypothesis that cancer, in many instances, may be initiated specifically from a set of cells that have already undergone extensive epigenetic changes.^30^ Thus, analysis of the targets for altered DNA methylation that are conserved across all ALL subtypes may be able to identify key drivers of the disease that could be targets for the development of novel therapeutic approaches.

The cytogenetic subtype specificity of many of the methylation changes suggests that these differential methylation patterns may be important in the different biological/clinical behavior of the different cytogenetic groups. Here we used gene enrichment pathway analysis to investigate whether subtype specific DMCs might be preferentially targeting specific biological pathways. Our observation of gene enrichment pathways in *ETV6-RUNX1* involving immunity and infection pathways (8/10 pathways with a significant association are related to immune function) is a potentially exciting avenue for future analysis, as it may relate to previous reports that have suggested abnormal immune response as a major factor shaping the trajectory of leukemogenesis.^31,32^ In the *TCF3-PBX1* subtype, we identified a remarkable enrichment for genes in the neuroactive ligand-receptor interaction pathway. Interestingly, a large fraction of these methylation changes map to gene promoter regions, suggesting that they are likely to be associated with functional changes in gene expression. Previous reports have shown that neuroactive ligand-receptor interaction pathways are associated with acute leukemias as well as several other diseases^33,34^ Further investigation of the potential role of this pathway in *TCF3-PBX1* driven leukemia would be warranted. Integrating the methylation data with gene expression data would help clarify whether the subtype specific patterns of methylation correlate with clear differences in subtype specific gene expression and thus potentially with downstream gene function.

This study focused specifically on DNA methylation. To more clearly understand the epigenome of pediatric ALL it may be necessary to undertake integrative studies assessing other epigenetic mechanisms, such as nucleosome remodeling and histone modifications, as well as associations with microRNA and gene expression in the same sample sets. In addition, while the Infinium HumanMethylation450 BeadChip array platform used in this and other studies^20^ has coverage of much of the genome, including 99% of RefSeq genes, it only contains probes for about 2% of the total number of CpG sites in the human genome. Some studies have used whole-genome bisulfite sequencing in an attempt to address this limitation. However, at this time, it is challenging to apply this approach to more than just a small number of samples due to cost and increased DNA requirement.^35-37^. Thus, further technical developments in genome-wide bisulfite sequencing and integration with other epigenetic mechanisms will be required to allow the identification of a complete picture of the epigenetic changes in childhood ALL and how this relates to changes in gene expression profiles. Such integrative studies may reveal biologically relevant epigenetic changes.

## Materials and Methods

### Patients and sorted cells

Bone marrow samples from 52 pediatric patients with *ETV6-RUNX1* (n = 20), high hyperdiploidy (51–65 chromosomes) (HeH) (n = 20), *TCF3-PBX1* (n = 6) and dic(9;20)(p11–13;q11) (n = 6) who consented to be enrolled on the ethically-approved UK ALL treatment trial, ALL97/99. All samples used had a high blast count (average 93%). An additional 123 diagnostic bone marrow samples from MRC ALL97/99 (HeH, n = 66 and *ETV6-RUNX1*, n = 57) were used in confirmatory pyrosequencing analysis.

### MLPA

Genomic DNA from patient bone marrow aspirates was extracted using standard procedures. Genomic DNA from healthy donors was obtained for use as control samples. DNA was analyzed using the SALSA MLPA Kit P335 (MRC Holland, Amsterdam, The Netherlands), as described previously.^25^ This kit includes probes for *IKZF1* (8 probes), *CDKN2A/B* (3 probes), *PAX5* (6 probes), *EBF1* (4 probes), *ETV6* (6 probes), *BTG1* (4 probes), *RB1* (5 probes), and the PAR1 region: *CRLF2, CSF2RA*, and *IL3RA* (one probe each). Data were analyzed using GeneMarker V1.85 analysis software (SoftGenetics). All loci were found to be deleted in at least one patient and the majority of patients (40/52) had deletion of one or more of the genes assessed.

### Bisulfite conversion and 450K array hybridization

Bisulfite conversion was performed using the Zymo EZ-96 DNA methylation kit and the bisulfite converted DNA was hybridized to the HumanMethylation450 Analysis BeadChip (Illumina) and processed following the 450K methylation array procedure, according to manufacturer's instructions. Hybridization fluorescent signals were read by the Illumina BeadStation GX scanner. This procedure was performed at Wellcome Trust Clinical Research Facility, Edinburgh, UK.

### Bioinformatics analyses

The arrays report DNA methylation status (β value) at > 485,000 CpG loci. The β value can range from 0 to 1, representing fully unmethylated and methylated values. Array processing, normalization and quality control checks, as well as derivation of the β values from the raw intensity values (.idat files), were implemented using the R package ‘minfi’.^38^ We employed conservative quality control measures to filter out poorly performing and potentially confounding loci. Briefly, a filtering process removed unannotated probes (i.e., not mapped to the genome), probes located on chromosomes X/Y, probes that aligned to more than one place in the genome, allowing for 1 mismatch, and probes that had a SNP with a minor allele frequency of 5% or greater within 50 bp of the interrogated site. Probes that failed in >5% of samples were also removed.^39-42^ The remaining probe β values (429,750) were converted to M-scores,^43^ and the top 10,000 most variable probes by standard deviation were selected for subgroup identification. The 10,000 most variable probes were used for the clustering as this is equivalent to the inflection point in the curve (when mapping variability versus probe number), such that probes that were excluded were largely non-variable and would have added little to the clustering. A non-negative matrix factorization (NMF),^44^ based consensus clustering approach was performed using the R package NMF.^45^ An optimal factor number (i.e., subgroup number) was selected by maximizing cluster number while maintaining cophenetic correlation coefficient. Cluster stability measures (silhouette scores) were used to assess the quality of the identified subgroups. Pathway enrichment analysis was performed using ‘GOseq’ bioconductor package using the KEGG database. *P*-values were calculated by using both resampling and the Wallenius approximation based methods available in GOseq.^46^ *P*-values were also adjusted to control the false discovery rate (FDR) using the Benjamini–Hochberg method. The list of genes associated with probes was derived from the annotation provided by Illumina. The DNA methylation dataset is available at the Gene Expression Omnibus (GEO) with accession number GSE69229.

### Quantitative DNA methylation analysis using pyrosequencing

Genomic DNA (200 ng) was modified with sodium bisulfite using the Methylamp™ One-Step DNA Modification Kit (Epigentek, Brooklyn, NY, USA) as per the manufacturer's instructions. All samples were re-suspended in 15 μl of TE, and 1 μl of this was used for subsequent PCR reactions. DNA samples were amplified in 25 μl volumes containing 1X manufacturer's buffer, 1 unit of FastStart Taq polymerase (Roche, Welwyn Garden City, UK), 1–4 mM MgCl_2_, 10 mM dNTPs, and 75 ng of each primer. PCR was performed for 40 cycles with an annealing temperature of 53–63°C, depending on the primer set being used. For each set of primers (listed in Supplementary **Table 3**) one of the forward or reverse primers included a 5′ biotin label to allow for subsequent analysis by pyrosequencing. Following PCR amplification, sequencing was performed using a PSQ 96MA pyrosequencer (Qiagen, Hilden, Germany), as per manufacturer's protocol. For all loci, assays were performed in duplicate and values averaged between the duplicates. Only samples that were passed by the pyrosequencer were included and to further ensure a high degree of accuracy only runs in which single peak heights were in excess of 200 were included. For CpG island loci, between 3 and 6 consecutive CpG sites were measured and the methylation value for each locus was taken as the mean of all CpG sites measured at that locus. For non CpG island associated CpG sites, pyrosequencing assays were designed to include that single specific CpG site. Primer design was performed using the manufacturer's provided PyroMark software and all pyrosequencing runs included *in vitro* methylated DNA (Millipore, Watford, UK) and normal peripheral blood derived DNA as control.
