# Supplementary material for: Epigenetic landscape correlates with genetic subtype but does not predict outcome in childhood acute lymphoblastic leukemia
Source: Epigenetics. 2015 Aug 3;10(8):717–26. doi: 10.1080/15592294.2015.1061174 (PMC4622588; doi:10.1080/15592294.2015.1061174)
Supplement: Supplemental_Material.zip [file kepi-10-08-1061174-s001.zip › Supplemental Table Captions.docx]

Table S1. Subtypes specific DMCs.

Table S2. KEGG pathway analysis for subtype specific DMCs.

Table S3. Primers used for pyrosequencing analysis.
