# Supplementary material for: Epigenetic landscape correlates with genetic subtype but does not predict outcome in childhood acute lymphoblastic leukemia
Source: Epigenetics. 2015 Aug 3;10(8):717–26. doi: 10.1080/15592294.2015.1061174 (PMC4622588; doi:10.1080/15592294.2015.1061174)
Supplement: Supplemental_Material.zip [file kepi-10-08-1061174-s001.zip › Supplemental Material-Figures.doc]

**Fig. S1**. The optimal metagene number was selected by maximizing cophenetic coefficient and average silhouette score at each factorization. The optimally-performing four metagenes showed close relationships to the pre-existing genetic subgroups (Fig. 1), but not with diagnostic and relapse status.

**Fig. S2.** Genomic distribution of DMCs. A) Number of DMCs in promoter vs. non-promoter regions. B) Proportion of DMCs that are hypomethylated vs hypermethylated. C) Number of DMCs in CpG vs. non-CpG islands. D) DMCs location. E) DMC locations in relation to CpG sites

**Fig. S3.** Gene copy number generated from raw methylation probe intensity values, using previously published methods ^43^. Leukemic sample intensities were compared against 27 normal blood samples, downloaded from the Gene Expression Omnibus dataset GSE41169 ^44^. Magenta vertical lines mark probes +/- 100kb from gene location A) Normal patient for *PAX5* on chromosome 9 with two copies of *PAX5*. B-F) *PAX5* deletion in *ETV6-RUNX1* positive patients previously identified by MLPA The blue horizontal line is a loess line plotted across log ratios, and is indicative of underlying copy number status.

Fig. S4. Replication of methylation levels using pyrosequencing. To confirm the accuracy of the methylation levels derived from the array analysis, nine loci were additionally assessed using pyrosequencing. These loci consisted of 3 promoter associated CpG islands for which previous preliminary data had suggested a potential association with outcome (*HOXA4* ^41^, *TWIST2* ^42^ and *TUSC3* (unpublished data)), 3 additional promoter-associated CpG islands (at the *KCNA3*, *EXT1* and *C15orf14* loci) and three individual CpG sites located outside CpG islands (within the *BTBD9*, *Gelsolin* and *RNF6* loci). Loci were chosen on the basis of limited, non-significant (with the exception of *EXT1*), association with outcome and variability in methylation levels across the sample set. Examples shown for A: *TWIST2* and B: *TUSC3*, both of which showed a very high degree of agreement between the array and pyrosequencing analysis. C: R values (Pearson correlation coefficient) for correlations at all nine loci assessed by pyrosequencing, shows highly significant correlations between pyrosequencing and array analysis. Analysis was carried out for CpG sites within CpG islands (six loci, comparing average values from 3-6 CpG sites covered by pyrosequencing assays to average of all CpG sites within the corresponding CpG island included on the array (CpG island)) or for specific CpG sites (three loci, comparing pyrosequencing and array values at one specific CpG site (single)). D: Pyrosequencing analysis of CpG site mapping to north shore of the *KCNA3* locus in *ETV6-RUNX1* associated cases. This confirms the array data identifying low methylation in diagnostic *ETV6-RUNX1* associated cases without a subsequent relapse (NR (array)) and higher levels in diagnostic samples which did subsequently relapse (R (array)). However analysis of an additional 57 *ETV6-RUNX1* associated cases, not included in the array study, failed to confirm this association with outcome. E: Assessment of methylation levels using pyrosequencing for the EXT1 locus. Pyrosequencing analysis of 5 CpG sites mapping to promoter associated CpG island in the EXT1 locus in HeH associated cases. This confirms the array data identifying low methylation in diagnostic HeH associated cases without a subsequent relapse (NR (array)) and higher levels in diagnostic HeH samples which did subsequently relapse (R (array)). However analysis of an additional 66 HeH associated cases, not included in the array study, failed to confirm this association.
